# A Review of Essential Oils with Anti-*Campylobacter jejuni* Effects—Their Inhibitory and Destructive Effects on Biofilms and Efficacies on Food Matrices

**DOI:** 10.3390/foods15030471

**Published:** 2026-01-29

**Authors:** Anita Seres-Steinbach, Krisztián Bányai, György Schneider

**Affiliations:** 1 Department of Medical Microbiology and Immunology, Medical School, University of Pécs, 7624 Pécs, Hungary; 2 Department of Medical Biology, Medical School, University of Pécs, 7624 Pécs, Hungary; 3 Department of Pharmacology and Toxicology, University of Veterinary Medicine, 1078 Budapest, Hungary

**Keywords:** *Campylobacter jejuni*, essential oils, preservation

## Abstract

*Campylobacter jejuni* is an important foodborne pathogen. To prevent human infections, special attention should be paid to prevention. Recently, methods involving essential oils have been considered as a means of reducing the number of contaminants in and on foods. This review summarizes the results of studies in which essential oils (EOs) with anti-campylobacter effects were tested. The most widely studied EOs were clove (28%), oregano (24%), thyme (22%), rosemary (8%), lavender (7%), sage (7%), and tea tree (4%), with other EOs studied to a lesser extent. The anti-Campylobacter efficacies of these EOs were demonstrated in vitro using a broad repertoire of methods, such as minimal inhibitory and bactericidal concentrations, agar diffusion, time-kill assays, adhesion and biofilm inhibitory assays, two-dimensional polyacrylamide gel electrophoresis, quantitative reverse-transcription PCR, and liquid chromatography–mass spectrometry. Recent studies have also focused on the practical application of such EOs, with experiments performed on different food matrices, typically chicken, duck, and beef. The most frequent treatment methods were mixing, dipping, and short-time freezing, either in packed or unpacked forms, and storage at different temperatures (typically 4 °C), although experiments were also performed at 25 °C, 32 °C, and 42 °C using different EO concentrations. In summary, these experiments revealed the anti-Campylobacter effects of thyme, cinnamon, coriander, lime, oregano, chrysanthemum, and basil.

## 1. Introduction to *Campylobacter jejuni*—A Foodborne Pathogen

Campylobacteriosis is one of the four main global causes of diarrheal disease worldwide [1,2]. The most frequently reported species is *Campylobacter jejuni.* In some cases, infections can be asymptomatic, but specific or non-specific symptoms may appear after an incubation period of 1–7 days [3,4]. Non-specific symptoms include fever and headache while specific symptoms include diarrhea lasting 2–5 days and abdominal cramps and pain [5]. Sometimes, bloody stools are also present [6]. Complications resulting from the infection include cholecystitis (gallbladder inflammation) and pancreatitis (inflammation of the pancreas). The most significant post-infection complications are Guillain-Barré syndrome (GBS) and Miller Fisher syndrome (MFS), which affect the peripheral nervous system and occur in 1–2 people per 100.000 both in the United States [7,8] and in Europe [9]. An important public health concern is the emergence and spread of multidrug-resistant clones that require prolonged treatment and increased costs [10].

The economic burden of *C. jejuni* infections is around EUR 2.4 billion per year in the EU alone due to the increased health costs and losses in productivity [11]. In the United States, this sum is estimated to be between USD 1.3 and 6.8 billion [12].

In terms of the estimated number of infections worldwide, this is between 400 and 500 million cases per year [13,14]. From among the contributing factors that support this high number is the low infectious dose that means that just 500–10,000 bacterium cells are enough to colonize the gut and thereby cause an infection in humans [15,16]. This can be influenced by the susceptibilities of both the bacterium strain and the host.

*C. jejuni* commonly colonizes the digestive tract in chickens, and since the consumption of poultry has been permanently increased over the last three decades, this means a risk factor. The prevalence of *Campylobacter* species in poultry, particularly in broiler flocks close to slaughter age, can be as high as 100% [17], and the bacteria can spread easily within a flock via the fecal–oral route [18]. A survey carried out in Benin, West Africa, in 2020 found that the prevalence of *C. jejuni* was 23.4% [19]. Another study, conducted in metropolitan Accra, Ghana in 2022, found a *C. jejuni* prevalence of 38.3% in retail chicken meat [20]. However, alongside poultry, the consumption of raw milk, improperly pasteurized dairy products, and unwashed vegetables and fruits can also be a source of human infection [21,22]. Natural waters, such as lakes and rivers, can also act as reservoirs for infection if contaminated by wild animals or farm sewage and grey water [23,24]. Consuming such water can lead to campylobacteriosis [25,26]. It has recently been reported that flies can act as mechanical vectors, spreading *Campylobacter* and transferring it to food or even broiler houses. Therefore, protection against flies, rodents, wild birds, and other animals plays a key role in avoiding campylobacteriosis [27].

Although campylobacteriosis is usually a self-limiting infection, the abovementioned potential human health implications and economic burdens mean that the importance of prevention cannot be neglected. One of the most logical approaches is to remove this bacterium from the food chain and, by doing that, to prevent it from reaching consumers. Appropriate food safety measures such as maintaining cleanliness and hygiene in slaughterhouses, disinfecting carcasses, and ensuring clean eggshells can address this issue [28,29].

Several poultry vaccines have been developed over the years to suppress the spread of *C. jejuni* in chickens, including whole-cell [30,31], subunit [32,33], microorganism-vectored [34,35], and nanoparticle vaccines [33]. However, these have not be proven to be effective enough. Another potential solution is the oral administration of bacteriophages to broiler chickens, which, in certain cases, can significantly reduce the number of living *C. jejuni* before slaughter. Probiotic bacteria have also been shown to eliminate *C. jejuni* from the guts of animals [36].

To prevent *Campylobacter* infection during poultry processing, sprays and washes containing chlorine, chlorine dioxide, acidified sodium chlorite, trisodium phosphate, and peroxyacid are most commonly used [37,38]. Other physical disinfection methods used to eradicate the pathogen from meat surfaces include ozonation [39], irradiation [40], forced air chilling [41], steam pasteurization [42], steam-ultrasound [43], and freezing [44].

Due to their antimicrobial properties, essential oils have recently been considered as potential disinfectants, replacing chemical preservatives. In this review, we summarize the results of relevant in vitro anti-campylobacter tests, including anti-adhesion and antibiofilm tests, and present studies in which anti-Campylobacter effects were revealed in different food matrices.

## 2. Essential Oils: Their Antimicrobial Effects and Modes of Action

Essential oils are herbal extracts obtained by distilling various parts of plants, such as their roots, stems, leaves, and flowers. The biological activities of EOs have recently been shown to range across a wide spectrum, including neurological, antitumor, digestive, wound-healing, anti-inflammatory, antiparasitic, antiviral, and antibacterial properties [45]. These effects are influenced by the chemical compositions of oils, which are typically dominated by a few major compounds rather than a greater number of minor ones. The chemical compositions of the essential oils can be affected by various internal and external factors, including the plant’s physiological state and environmental conditions such as the altitude, rainfall, number of light hours, soil quality, mineral content, and, of course, extraction technique employed [46,47,48]. The most important extraction methods are cold pressing, steam distillation, solvent extraction, supercritical fluid extraction, and microwave separation [49]. All these factors determine the biological activities of EOs.

The quantitative and qualitative features of these compositions determine whether an essential oil has an antibacterial effect on a bacterium. From this perspective, the most significant compound groups are terpenes and terpenoids, phenylpropanoids, aldehydes, ketones, alcohols, esters and oxides [50]. In terms of antimicrobial efficacy, terpenes are probably the most significant group. Multi-component monoterpenes consist of terpenes and sesquiterpenes, which can have aromatic consequences. The variability of these compounds is further increased by the presence or absence of different functional groups, which can strongly influence the biological effects [51]. Previous studies have shown that the primary mode of action of the hydrophobic compounds of EOs is membrane damage. During this process, the compounds bind to the apolar part of the cell membrane—specifically, the phospholipids—via London dispersion forces. Consequently, the cell membrane becomes permeable and soluble, and ions begin to flow out of the bacterial cell. A damaged membrane cannot perform basic functions, and due to the equalization of the pH gradient, ATP synthesis becomes impaired [52,53].

The number of studies demonstrating that different chemical variants of certain compounds can directly impact specific stages of bacterial metabolism is growing. While the mechanism of action of many components remains unknown, it is known that carvacrol and thymol affect membrane permeability, and the hydroxyl group of eugenol can bind to proteins, thus ensuring a specific effect. Essential oils can affect enzyme function: for example, some ketones and alcohols act as acetylcholinesterase inhibitors (e.g., isomenthone and menthone) while d-carvone inhibits glutathione S-transferase activity. Tyrosinase has also been shown to be a polyphenol oxidase inhibitor. Tyrosinase has also been shown to be inhibited with citronella. Furthermore, EOs can affect the functioning of enzymes involved in energy regulation and the synthesis of structural components [54,55].

It has also been reported that the position of the hydroxyl group affects a component’s effectiveness. The aldehyde group, which is typically present in *Melissa officinalis* (citral) and *Eucalyptus citridora* (citronellal), is strongly electron-negative, affecting antimicrobial ability by interfering with physiological processes involving electron transfer. It can react with nitrogen and thereby inhibit the growth of microorganisms. An interesting observation based at least on the available data is that alpha isomers are less active than beta isomers [56].

## 3. Safety of Essential Oils as Food Ingredients

Using essential oils and their components in food poses challenges as many questions arise regarding safety. Since 1996, researchers have been interested in how to use essential oils safely. Nowadays, an increasing number of experiments are investigating the genotoxicity and cytotoxicity of essential oils. In 2017, experiments were conducted on human HEL 12,469 cells using six essential oils. It was revealed that none of the oils (oregano, thyme, clove, lavender, clary sage, and arborvitae) exhibited significant genotoxicity [57]. In another experiment, human lymphocytes were exposed to various concentrations of tea-tree essential oil. None of the following concentrations affected lymphocyte morphology: 95 μg/mL, 182 μg/mL, and 365 μg/mL [58]. Dill, peppermint, and pine essential oils were also tested for genotoxicity in human lymphocytes in vitro, and in a *Drosophila melanogaster* somatic mutation and recombination test (SMART), which includes chromosome aberration (CA) and sister chromatid exchange (SCE) tests. Of these essential oils, dill seed essential oil was almost inactive in the SMART test, whereas peppermint essential oil exerted a mutagenic effect regardless of the dose administered. Unfortunately, all of the essential oils tested showed chromosomal abnormalities in human lymphocytes [59]. The genotoxicity of *Salvia officinalis* was also investigated in both mouse bone marrow and male germ cells. The essential oil was compared to CCL_4_. Under the experimental conditions, it was found to be non-genotoxic, and it was even able to modify genetic damage when combined with CCl_4_ [60]. Given the prevalence of articles on the use of oregano essential oil, it is no surprise that Ipek and his colleagues investigated its genotoxicity and antigenotoxicity. During their research, they determined that carvacrol, a component of oregano oil, may have a significant pharmacological role in the future, potentially in cancer therapy, given its antimutagenic properties [61]. Another study also examined the genotoxicity of oregano, and concluded that it has strong antioxidant and antigenotoxic effects [62]. Rosemary is a popular spice that is commonly used. It is also frequently used against foodborne pathogens. No genotoxicity was observed under the study conditions, and it was revealed that rosemary can prevent oxidative DNA damage in human lymphocytes [63]. Furthermore, the active ingredient of basil, linalool, has been shown to be antigenotoxic and to have antioxidant properties [64,65]. Based on these examples, it is important to test potential essential oils for genotoxicity before considering their practical use.

Determining the applied EO concentrations is crucial not only for efficacy but also for safety. Based on the results of some recent studies, the generally effective concentration of EOs is around 2%. However, this value depends on the method of application, such as direct pickling, spraying, immersion, or steaming [66]. Higher concentrations may affect the sensory properties of meat and are therefore not preferred for fresh meat or any other type of food [67]. Many essential oils (e.g., oregano, thyme, cinnamon, and basil) are listed as GRAS (Generally Recognised as Safe) by the FDA—meaning that they are considered safe for use in food in small quantities [68]. However, the concentration and biodistribution of ingredients after absorption may differ significantly from their action on microorganisms during contact with the meat surface. Therefore, while GRAS status essentially indicates safety, it does not mean that all essential oil components are safe for the human body at high concentrations. This feature is characterized by the safety margin window. Ideally, the antibacterial concentration should be much lower than the concentration at which damage to eukaryotic cells occurs [69]. From this perspective, oregano, thyme, clove, and cinnamon are ideal, but tea tree, eucalyptus, and rosemary, or EOs with a high terpenoid content, are less ideal [43,70,71,72,73,74].

## 4. Methods to Reveal the Anti-Campylobacter Activity of Essential Oils and Their Active Components

Due to their beneficial properties, such as their antibacterial and antioxidant effects, essential oils have been considered as potential food preservatives since the 1980s. They can be used to extend the shelf life of various foods and reduce the number of pathogenic bacteria on and in food samples. Essential oils can be screened for anti-Campylobacter activity using both inexpensive, cost-effective methods as well as more complex and expensive ones.

The most widely accepted and simplest antibacterial-effect-screening methods are the drop plate method, the agar diffusion method, and the disk diffusion method. In the drop plate method, the essential oils to be tested are dropped onto a lawn of the target bacterium. However, some discrepancies can be found among the articles as the volume of essential oils applied to the bacterial lawn plays an important role in the size of the inhibition zones. The inhibition zone of thyme on *C. jejuni* measured 13.3 ± 1.7 mm at 2.5 µL, increased to 15.5 ± 1.0 mm at 5 µL, and further grew to 20.4 ± 1.2 mm at 10 µL and 28.7 ± 2.1 mm at 20 µL. The inhibition zone of oregano measured 14.4 ± 1.2 mm at 2.5 µL, increased to 19.8 ± 1.9 mm at 5 µL, and further grew to 24.5 ± 4.6 mm at 10 µL and 28.5 ± 2.4 mm at 20 µL. Generally, the larger the volume of essential oil applied is, the larger the inhibition zone size will be [75]. In the case of the agar diffusion method, the sample is transferred into an agar hole containing a properly diluted bacterial suspension. In the disk diffusion method, a filter paper disk soaked with essential oil is placed on the bacterial lawn. In all three cases, the results are evaluated after the incubation period (12–24 h).

Once an essential oil has been found to be effective, its minimum inhibitory concentration (MIC) and the minimum bactericidal concentration (MBC) can be determined in a liquid medium using micro- or macrodilution techniques. This procedure is very similar to the method of MIC and MBC determination for antibiotics. Ideally, however, the suspension of EOs in a hydrophilic liquid medium would be facilitated using detergents (e.g., Tween 20). Different concentrations of essential oils are then added to the broth containing CFU-synchronized bacterial suspensions (see Table 1).

A time-killing assay reveals and compares the antibacterial kinetics of the investigated EOs at different concentrations over time [52,76]. During the procedure, the number of pathogens can be tracked in the presence of the different essential oils. The inflection point of the curve at certain concentrations indicates the duration of the antimicrobial effect. Morphological changes in EO-exposed *C. jejuni* cells can be revealed by using microscopic techniques; scanning electron microscopy (SEM) is one of the most spectacular. This method can visualize changes in shape. In the case of *C. jejuni*, the most important characteristic visible changes are twisting and alterations in length, but rounding may also be observed when the bacterial cell transforms into a viable but non-culturable form, as was recently described in the presence of peppermint EO. *C. jejuni* cells became straightened and elongated [52,53] while cells exposed to clove oil became straightened but shortened [52]. Exposure to higher concentrations resulted in a complete destruction of *C. jejuni* cells, which appeared as cell ghosts [52,53].

Morphological changes refer to molecular occurrences that affect different metabolic functions such as the involvement of the cytoskeletal system. Methods such as transcriptomics and proteomics are useful for identifying whether these occurrences affect transcriptomic or protein synthesis levels. Whole transcriptome analysis (WTA) reveals global changes represented by the activities of the genome’s transcripts while quantitative reverse-transcription PCR (qRT-PCR) can study the expression changes of selected target genes in detail [77,78]. Comparing transcriptomic data with protein profiling results can provide additional insight, particularly when combined with WTA results. The aforementioned elongated morphological changes were associated with stress responses that could affected the peptidoglycan modifying enzyme Pgp1, which is required for the helical shape [52,53,79,80,81].

Biofilm formation is a crucial aspect of bacterial survival on both biotic and abiotic surfaces [82,83]. The ability of the isolates to form biofilms is most frequently demonstrated using 96-well polystyrene plates. The formed cell matrix can be visualized using crystal violet staining, whereby the intensity of the stain is directly proportional to the amount of biofilm formed [84,85]. Despite *C. jejuni*‘s ability to form biofilm causing problems in the food industry during processing at lower temperatures (14–25 °C), many experiments are still carried out only at higher temperatures (37 °C), where biofilm formation is less relevant [86,87,88,89]. Nevertheless, if biofilms are already present, testing the inhibitory and degradative potential of essential oils is reasonable as this is practically relevant.

## 5. Essential Oils with Anti-Campylobacter Activities

**Figure 1:**
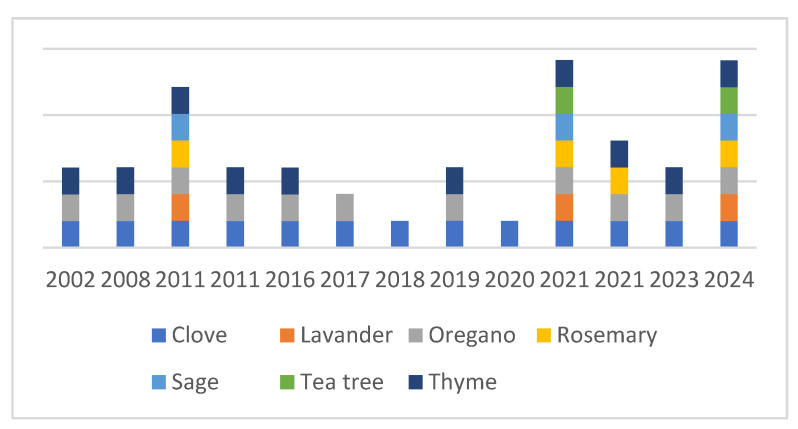
Percentage comparison of the publications focusing on the anti-Campylobacter effects on various essential oils from WHO [1].

The number of experiments investigating the antimicrobial effects of essential oils on *C. jejuni* has increased since the 1990s. Due to their general antibacterial properties and potential practical relevance, different authors, such as Salem et al. (2019) [90], have screened the anti-Campylobacter effects of various essential oils, including thyme, cinnamon, clove, and oregano [52,88,91,92]. When conducting a PubMed search for *C. jejuni* and various essential oils, it becomes clear that the number of EOs tested against *C. jejuni* is still fairly limited (Figure 1).

The antibacterial effect of essential oils is mostly attributed to their major components. For example, in the case of cinnamon, the major component is cinnamaldehyde [93]; in the case of thyme, the major antibacterial component is a-terpinene and the minor component is I-terpineol [94].

### 5.1. Thyme

Due to its antibacterial properties, thyme (*Thymus vulgaris*) is a promising EO for practical purposes, and thus, several studies have investigated this EO. Babu et al. reported an inhibition zone size of 13 ± 0.06 mm whereas Salem et al. measured zones ranging from 20 to 28 mm [91]. Mutlu-Ingok et al. conducted their experiments on multiple strains and observed a strong strain-dependent effect with thyme, measuring inhibition zones at 20.4 ± 1.2 mm [75]. To improve efficacy, thyme was tested in combination with orange oil. The results showed that the large inhibition zone originally caused by thyme was reduced to a diameter of 22 mm while the inhibition zone of orange essential oil increased from 16–18 mm to 22 mm. Sterile filter paper disks (6.0 mm in diameter) were impregnated with 10 µL of either undiluted individual essential oils or a thyme–orange combination (TOC), consisting of 5 µL thyme oil and 5 µL of orange oil, and were evaluated 48 h after incubation in microaerobic conditions at 42 °C [95]. The minimum inhibitory concentration (MIC) of thyme was reported to be 4.000 µg/mL [92] while no minimum bactericidal concentration (MBC) was determined, similar to the study by Mutlu-İnğok et al. (2021), where MIC and MBC values were 21.61 μg/mL [75].

### 5.2. Oregano

Similarly to thyme, oregano is a widely used essential oil. In terms of individual inhibition zones, Aslim and Yucel (2008) [96] (*Origanum minutiflorum*) measured the largest zones against ciprofloxacin-resistant *Campylobacter* spp. (twelve *C. jejuni*, five *C. lari*, and four *C. coli*). This is important because antibiotic resistance is both a food safety and public health issue; however, these results further reinforce that essential oils can provide an alternative solution even against antibiotic-resistant strains. The inhibition zone for *C. jejuni* ranged from 12 to 27 mm (1/10 diluted with ethanol) or 9 to 28 mm (1/15 diluted with ethanol). Pesavento et al. (2015) (*Origanum vulgare*) [92] reported zones ranging from 14 to 18 mm when 0.5% Dimethyl Sulfoxide was added to a final volume of 2 mL at 25%, 50%, 75%, 100% (*v*/*v*). They used two different strains, and they used sterile filter paper disks measuring 6 mm in diameter, which were soaked with 10 μL of EO dilution. In another study [75], zones ranging from 13.3 to 28.7 mm were measured. Their EO suspensions were made with 10% DMSO, and they also used 6 mm sterile disks soaked with 2.5–20 μL of EO [75]. El Baaboua et al. (2022) [88] measured inhibition zones ranging from 15 to 80 mm for *Origanum compactum Benth*. For these tests, sterile filter paper disks (6.0 mm in diameter) were impregnated with 50 µL of undiluted EO. The filter paper disks were impregnated with essential oils of different concentrations; however, it was clearly visible that the higher the concentration of the essential oil was, the larger the measurable inhibition zone was. In the case of undiluted oil, inhibition zones of up to 80 mm were observed, while for diluted oil, the largest inhibition zone measured in this study was 28 mm.

None of the research groups reported MBC values while the MIC values were as follows—El Baaboua et al. (2022) [88]: (*Origanum compactum Benth*) 592–1175 µg/mL, Mutlu-Ingok et al. (2021) [75]: (*Origanum vulgare*) 5.65 µg/mL, and Aslim & Yucel (2008) [96]: (*Origanum minutiflorum*) 12.5–700 µg/mL. The relatively large differences between the MIC values can be attributed to the different species of oregano used, as well as the fact that the researchers tested susceptibility on different bacterial strains.

### 5.3. Cinnamon

Babu et al. (2011) [91] studied the efficacy of cinnamon essential oil on a reference strain of *C. jejuni*, revealing an inhibition zone of 26.00 ± 0.06 mm when the oil was diluted in diethyl ether. They used the agar diffusion assay to determine the MIC [91].

Gahamanyi and colleagues (2020) [97] investigated the MIC and MBC values of cinnamon essential oil (*Cinnamomum zeylanicum*). They found that *C. jejuni* susceptibility to the essential oil could be influenced by whether the bacterium was isolated from a chicken or from a human patient. The MIC and MBC values for the *C. jejuni* strain isolated from chicken were both 25 µg/mL; however, the strain isolated from the patient had an MIC value of 25 µg/mL and an MBC value of 50 µg/mL. Subsequently, they examined the main component of the oil, (E)-cinnamaldehyde, and found that its MIC and MBC values were identical—exactly the same as those of the cinnamon essential oil [97].

Meanwhile Pesavento e al. (2015) [92] reported that the MIC value of cinnamon essential oil was 2500 µg/mL, with an inhibition zone ranging from 13.7 to 20.3 mm depending on the applied concentration. Disk diffusion was used with 10 µL of the essential oil dropped onto 6 mm filter paper disks [92].

### 5.4. Clove

Due to its effective antibacterial properties, clove oil (derived from *Eugenia caryophyllata*, or *Syzygium aromaticum*) is a commonly used essential oil tested against *C. jejuni*. With regard to the diameters of the inhibition zones on the *C. jejuni* lawns, the values were 13 ± 0.08 mm and 17.5 mm [91,95]. Both research groups used disk diffusion assays; however, one group used sterile filter paper disks (6.0 mm in diameter) soaked with 10 µL of individual undiluted essential oil [95] while the other group used sterile paper disks (Whatman, 1.6 mm) with 30 µL of absorbed essential oil [91]. In Kovács et al.’s (2016) [52] experiments, the MIC value was found to be 200 µg/mL, and the MBC value was found to be 800 µg/mL [52]. These values were similar to those found the MIC values of another study [98], which found the MIC values to range between 50 and 100 µg/mL, and also, the MBC values ranged between 50 and 100 µg/mL [98]. Elgamoudi and Korolik (2021) [99] found that the MIC value of clove EO ranged from 50 to 400 µg/mL. The MIC value was influenced by the susceptibility of various strains [99]. Clove EO was found to markedly straighten and shrink *C. jejuni* cells, affecting the cytoskeletal, capsule, adhesion, and other genes [52].

### 5.5. Peppermint

The above-described observation is all the more interesting because the effect of peppermint oil (*Mentha × piperita*) caused the cells to become straightened and elongated, highlighting differences in the molecular modes of actions of different EOs. The up- and downregulation of certain metabolic-related (*dnaK*, *groEL*, *groES*) and virulence-associated (*cheY*, *flhB*, *flgE*, *cadF*, *wlaB*, *porA*, *cbf2*) genes or regulators (*flab*, *flgB*, *flgE2*) were also revealed. The MIC value was 100 µg/mL while the MBC value was 400 µg/mL [53]. Similar virulence features affecting phenotypic effects were found in the cases of juniper and rosemary that could reduce cell adhesion to surfaces and alter surface structure function, such as that of the flagella [100].

### 5.6. Rosemary

Rosemary (*Rosmarinus officinalis*) typically formed an inhibition zone of 15–17 mm (sterile filter paper disks, 6.0 mm in diameter, soaked with 10 µL of individual, undiluted essential oil) [95], whereas in El Baaboua’s (2022) study, it was 14–70 mm (sterile paper disks, Whatman 1.6 mm, with 30 µL of EO absorbed) [88]. The MIC and MBC values were >20,800–2600 µg/mL, which, was comparable to the values of juniper (*Juniperus communis*) being 1000 µg/mL.

### 5.7. Lavender

Although thyme, clove, cinnamon, and oregano are the most frequently tested EOs against *C. jejuni*, recently, attention has also turned to lavender (*Lavandula × intermedia*), based on two studies published in 2021 and 2022 [87,89]. In both studies, experiments were conducted with both the pure essential oil and ethanolic extracts. The MIC values for *C. jejuni* generally ranged from 0.2 to 1 mg/mL. In El Baaboua’s (2022) study, however, the MIC value for lavender was 0.63 mg/mL [88].

### 5.8. Other Essential Oils

In addition to the aforementioned EOs, the efficacy of citrus fruit (*Inula graveolens*) [101], Bay Laurel (*Laurus nobilis*) [101], the Mastic tree (*Pistacia lentiscus*) [101], Manuka (*Leptospermum scoparium*) [102], and Lemon myrtle (*Backhousia citriodora*) [102] were reported against *C. jejuni*. However, these EOs have only been investigated superficially, and the available information is mainly based on the results of the most frequently used screening method: the disk diffusion method. The diameters of the inhibition zones, as determined with the drop plate method or with the agar disk diffusion assay, were generally between 11 and 90 mm. For citrus fruit EO, these values were between 18 and 23 mm [101]; for *Inula graveolens*, around 50 mm [101]; for *Laurus nobilis*, 37 mm [101]; and for *Pistacia lentiscus*, 25 mm [101]. The highest inhibition zones were found with *Leptospermum scoparium* (90 mm) [102] and *Lemon myrtle* (90 mm), both of which have citral as the major component [102]. Disks (6 mm) were placed on the surface of the agar and impregnated with 10 µL of the test agent. Leptospermum oil was diluted in PFG to obtain a concentration of 20% (*v*/*v*) whereas the other samples were tested undiluted [102]. In the agar-well diffusion method, three wells were cut out of the agar using a sterile cork borer and filled with 20 µL of essential oil [103]. To prepare the stock solutions of the samples, the pure essential oils were diluted in 5% (*v*/*v*) dimethyl sulfoxide (DMSO). Then, sterile filter paper disks (6 mm in diameter) were impregnated with 0.05 mL of EO using a capillary micropipette [101].

Cardamom oil is a less commonly used EO [103], with MIC and MBC values of 25 μ/mL and an inhibition zone measuring 24.75 ± 2.00 mm. These MIC and MBC values were found to be identical to those of dill essential oil (*Anethum graveolens*) [103], although dill showed a smaller inhibition zone. The MIC and MBC values for fennel essential oil were 28.5 mg/mL [75], and with 20 µL applied, the inhibition zone measured 17.9 ± 3.7 mm. An even smaller inhibition zone was observed for garlic (*Allium Sativum* L.) [91]. The anti-Campylobacter effects of the components of carrot EO, such as (E)-methylizoeugenol and elemicin, were tested found to have MIC values between 64 and 128 μg/mL [104]. Interestingly, the MIC of the EO was not presented.

### 5.9. Combined Effects of Essential Oils

Some recent studies have focused on the synergistic effects of cumin, cardamom, and dill seed. It was found that the essential oils were more efficient when used in combination than individually. The MIC value of cardamom (*Elettaria cardamomum* (L.) *Maton*) essential oil was found to be 0.025 µL/mL when used alone; however, this increased to 0.050 µL/mL when cardamom and cumin were applied together. Adding dill weed to cardamom decreased the MIC value to 0.012 µL/mL. However, when cardamom was combined with both cumin (*Cuminum cyminum* L.) and dill weed (*Anethum graveolens* L.) the MIC value matched the original value of 0.025 µL/mL [105]. In another study, the effects of thyme and oregano were investigated both individually and in combination. The results showed that thyme was more effective when used alone than in combination (no visible growth on the plate), whereas the effect of oregano was enhanced when used in combination. Oregano alone produced an inhibition zone of 16–18 mm, which increased to 22 mm when used in a mixture, while thyme’s effectiveness decreased in combination [95].

**Table 1:** Summary of the antibacterial tests performed with different essential oils on *C. jejuni* in recent studies. (Abbreviations: BHI: brain–heart infusion; MH: Mueller-Hinton; CCDA: Charcoal Cefoperazone Deoxycholate Agar; LB: Luria-Bertani; MIC: minimum inhibitory concentration; MBC: minimum bactericidal concentration; PAGE: polyacrylamide gel electrophoresis; TEM: transmission electron microscopy; SEM: scanning electron microscopy; PCR: polymerase chain reaction; qRT-PCR: quantitative reserve transcription polymerase chain reaction; FTIR: Fourier-Transform Infrared Spectroscopy; DPPH: 2,2-Diphenyl-1-picrylhydrazyl free radical assay; GC: gas chromatography; LC-MS: liquid chromatography–mass spectrometry; TLC-DB: Thin-Layer Chromatography–Direct Bioautography; AFM: atomic force microscopy; n.a.: no information is available).

Essential Oils	Aim of the Study	Applied Method	EO Compounds Investigated	Experimental Condition	MIC or Inhibition Zone (mm)	MBC	References
Basil (*Ocimum basillicum*)	Evaluation of twelve essential oils against *C. j.* in vitro and on food	Inhibition zone, MIC, time-kill assay	n.a.	BHI broth, beef, and chicken meat	10.4–13.5 ± 0.5 mm, 6.559–15.780 µg/mL	n.a.	[106] Rattanachaikunsopon (2010).
Bay (*Laurus nobilis*)	Pathogen reduction, lipid oxidation, and sensory freshness	Disk diffusion, MIC, sensory analysis	1.8-cineole, Terpenyl acetate	96-well plates, chicken meat	37.3 ± 5.5 mm 4.730 ± 395 µg/mL		[101] Djenane (2012).
Bergamot orange (*Citrus bergamia*)	To investigate the effectiveness of oils and vapors of EOs and their components against a number of common foodborne pathogens	MIC, survival inhibition area	Limonene, Linalool	Cabbage leaf, chicken skin	23 ± 0.3 mm	n.a.	[107] Fisher (2006).
Cardamom (*Elettaria cardamomum* (L.) *Maton*)	To determine how these essential oils kill or inhibit *Campylobacter* bacteria and understand the underlying mechanism, particularly how they damage the bacterial cell membrane	Agar diffusion assay, MIC, MBC, relative electric conductivity, extracellular ATP determination	α-terpinly acetate, 1.8-cineole	MH broth, CCDA medium	0.025 μL/mL, 24.75 ± 2.00 mm	0.025 μL/mL	[103] Mutlu-Ingok (2017).
Cinnamon (*Cinnamomum zeylanicum*)	To investigate how effective the EOs in spices are in inhibiting microbial growth and to determine the minimum concentration required to stop this growth	Disk diffusion, MIC	n.a.	BHI broth, Tryptic soy agar	26 + 0.06 mm	n.a.	[91] Babu (2011).
Cinnamon (*Cinnamomum zeylanicum*)	Evaluation of the antibacterial activity of 5 EOs in beef meatballs	Disk diffusion MIC, MBC, sensory analysis	Cinnamaldehyde	MH broth, Tryptic soy agar	230 μg/mL	230 μg/mL	[92] Pesavento (2015).
Cinnamon *Cinnamomum cassia* (L.)	Testing how well certain natural compounds and commonly used antibiotics can inhibit growth	MIC, MBC, PCR	n.a.	MH broth	200 µg/mL	400 µg/mL	[97] Gahamanyi (2020).
Chrysanthemum (*Chrysanthemum flos*)		Liposomes, FTIR, TEM	Complete EO	Chicken	n.a.	n.a.	[108] Lin (2019).
Clove (*Eugenia caryophyllata*)	EOs can break down *C. jejuni* biofilms, which could help prevent the spread of this foodborne pathogen	MIC	Complete EO	n.a.	50–400 μg/mL	n.a.	[99] Elgamoudi (2021).
Clove (*Eugenia caryophyllata*)	To test whether attaching antibodies to clove essential oil liposomes can make them more effective and longer-lasting in killing *C. jejuni*	Liposomes, time-kill assay, TEM	Complete EO	Chicken, beef	n.a.	n.a.	[76] Chen (2023).
Clove (*Eugenia caryophyllata*)	EOs can inhibit the growth of two important foodborne pathogens under laboratory conditions	Disk diffusion	n.a.	BHI broth, MH agar	No visible growth on the plate	n.a.	[95] Thanissery (2014).
Clove (*Eugenia caryophyllata*)	To investigate how effective the EOs in spices are in inhibiting microbial growth and to determine the minimum concentration required to stop this growth	Disk diffusion	n.a.	BHI broth, Tryptic soy agar	13 + 0.08 mm	n.a.	[91] Babu (2011).
Clove (*Eugenia caryophyllus*)	Evaluation of twelve essential oils against *C. j.* in vitro and on food.	Inhibition zone, MIC, time-kill assay	n.a.	BHI broth, beef, and chicken	20.2–23.6 ± 0.4 mm 473–1973 µg/mL	n.a.	[106] Rattanachaikunsopon (2010).
Clove (*Syzygium aromaticum*)	To evaluate the antimicrobial activity of clove essential oil and its effects on the virulence traits of *C. jejuni.*	MIC, MBC, time-kill assay, protein assay, SDS-PAGE, RT-PCR, SEM, motility assay, bioautography	Eugenol	CCDA plates, LB broth	200 µg/mL	800 µg/mL	[52] Kovács (2016).
Coriander (*Coriandrum sativum*)	Testing EOs at different conditions on *C. jejuni* in chicken burger and chicken shawerma	Inhibition zone	n.a.	Chicken, MH broth	no inhibition zone around the disk containing 1,2,3% coriander EO	n.a.	[109] Elsharawy (2018).
Coriander (*Coriandrum sativum*)	Testing whether certain essential oils can inhibit the growth of *C. jejuni*, a common cause of foodborne illness	Abiotic surface	n.a.	Chicken	n.a.	n.a.	[90] Salem (2019).
Coriander (*Coriandrum sativum*)	Evaluation of twelve essential oils against *C. j.* in vitro and on food.	Inhibition zone, MIC, time-kill assay	n.a.	BHI broth, beef, and chicken	23.3–26.8 ± 0.5 mm 276–552 µg/mL	n.a.	[106] Rattanachaikunsopon (2010).
Cumin (*Cuminum cyminum* L.)	To determine how these essential oils kill or inhibit *Campylobacter* bacteria and understand the underlying mechanism, particularly how they damage the bacterial cell membrane	Agar-well diff., MIC, MBC, relative electric conductivity, extracellular ATP determination	p-mentha-1.3-dien-7-al, Cumin-7-al, Cumin aldehyde, γ-terpinene, β- pinene	MH broth, CCDA medium	0.050 μL/mL, 19.75 ± 2.70 mm	0.050 μL/mL	[103] Mutlu-Ingok, (2017).
Dill (*Anethum graveolens* L.)	To determine how these essential oils kill or inhibit *Campylobacter* bacteria and understand the underlying mechanism, particularly how they damage the bacterial cell membrane	Agar-well diff., MIC, MBC, relative electric conductivity, extracellular ATP determination	Carvone, Limonene	MH broth, CCDA medium	0.025 μL/mL, 22.25 ± 1.60 mm	0.025 μL/mL	[103] Mutlu-Ingok (2017).
Elephant garlic (*Allium ampeloprasum*)	Evaluation of twelve essential oils against *C. j.* in vitro and on food.	Inhibition zone, MIC, time-kill assay	n.a.	BHI broth, beef, and chicken	16.2 ± 0.5 mm 1890–6030 µg/mL	n.a.	[106] Rattanachaikunsopon (2010).
Fingerroot (*Boesenbergia pandurata*)	Evaluation of twelve essential oils against *C. j.* in vitro and on food.	Inhibition zone, MIC, time-kill assay	n.a.	BHI broth, beef, and chicken	12.1–14.5 ± 0.7 mm 7.470–15.030 µg/mL	n.a.	[106] Rattanachaikunsopon (2010).
Fennel (*Foeniculum vulgare*)	Evaluating how this essential oils can fight harmful microbes and prevent oxidation, which is important for food safety and preservation	Inhibition zone, MIC, MBC	(*E*)-Anethole	Agar-well diff., antioxidant activity	28.5 μL/mL, 17.9 ± 3.7 mm (20 μL)	28.5 μL/mL	[75] Mutlu-Ingok (2021).
Garlic (*Allium Sativum* L.)	To test whether garlic essential oil can reduce or treat *Campylobacter*-induced illness under controlled experimental conditions	Colonization and translocation, histopathology	n.a.	*IL-10^−^* ^/^ *^−^* *mice*	n.a.	n.a.	[110] Heimesaat (2021).
Garlic (*Allium Sativum* L.)	To investigate how effective the EOs in spices are in inhibiting microbial growth and to determine the minimum concentration required to stop this growth	Disk diffusion, MIC	n.a.	BHI broth, Tryptic soy agar	13 + 0.13 mm	n.a.	[91] Babu (2011).
Garlic (*Allium Sativum*)	Evaluation of twelve essential oils against *C. j.* in vitro and on food.	Inhibition, MIC, time-kill assay	n.a.	BHI broth, beef, and chicken	14.3–18.2 ± 0.7 mm 2.250–9.000 µg/mL	n.a.	[106] Rattanachaikunsopon (2010).
Ginger (*Zingiber officinale*)	Evaluating how these essential oils can fight harmful microbes and prevent oxidation, which is important for food safety and preservation	MIC, FIC (synergetic)	α-Zingiberene, ar-Curcumene	Agar-well diff., antioxidant activity	6.577 μL/mL, 9.2–17.2 ± 2.6 mm	6.577 μL/mL	[75] Mutlu-Ingok (2021).
Greater galangal (*Alpinia galangal*)	Evaluation of twelve essential oils against *C. j.* in vitro and on food.	Inhibition zone, MIC, time-kill assay	n.a.	BHI broth, beef, and chicken	15.1–19.2 ± 0.3 mm 2.250–9.000 µg/mL	n.a.	[106] Rattanachaikunsopon (2010).
Holy basil (*Ocimum sanctum*)	Evaluation of twelve essential oils against *C. j.* in vitro and on food.	Inhibition zone, MIC, time-kill assay	n.a.	BHI broth, beef, and chicken	8.3–11.0 ± 0.3 mm 15,030–8000 µg/mL	n.a.	[106] Rattanachaikunsopon (2010).
Juniper (*Juniperus communis*)	EO can break down *C. jejuni* biofilms, which could help prevent the spread of this foodborne pathogen	MIC	n.a.	n.a.	1.000 µg/mL	n.a.	[99] Elgamoudi (2021).
Kaffir lime (*Citrus hystix*)	Evaluation of twelve essential oils against *C. j.* in vitro and on food.	Inhibition zone, MIC, time-kill assay	n.a.	BHI broth, beef, and chicken	10.5–13.1 ± 0.3 mm 9.000–18.000 µg/mL	n.a.	[106] Rattanachaikunsopon (2010).
Lavender (*Lavandula × intermedia*)	Chemically characterize, and evaluate the biofilm-control potential of lavandin essential oils and their distillation by-products	MIC, biosensor (intracellular signaling), adhesion, biofilm making, biofilm modulation, DPPH assay	Linalool, 1.8-Cineol, Terpinen-4-ol	Glass, 96-well plates	250–1.000 µg/mL	n.a.	[87] Ramić (2022).
Lavender (*Lavandula × intermedia*)	To chemically characterize and evaluate the biofilm-control potential of lavandin essential oils and their distillation to by-products	MIC, biosensor (intracellular signaling), adhesion, biofilm making, biofilm modulation, DPPH assay	Linalool, 1.8-Cineol, Terpinen-4-ol	Glass, 96-well plates	250–1.000 µg/mL	n.a.	[87] Ramić (2022).
Lavender (*Lavandula × intermedia*)	To chemically characterize and evaluate the biofilm-control potential of lavandin essential oils and their distillation to by-products	MIC, intracellular signaling, adhesion, biofilm modulation, DPPH assay	Linalool, 1.8-Cineol, Terpinen-4-ol	Glass, 96-well plates	250–1.000 µg/mL	n.a.	[87] Ramić (2022).
Lavender (*Lavandula stoechas* L.)	EO can inhibit resistant *Campylobacter* bacteria and prevent them from forming protective biofilms	Agar-well diffusion assay, MIC, MBC, biofilm	Fenchone, Camphor, Terpineol, Menthone	LB broth, 96-well polystyrene microtiter plate	2.350 μg/mL >80–48 mm	2.350 μg/mL	[88] El Baaboua (2022).
Lavender (*Lavandula × intermedia*)	EO can break down *C. jejuni* biofilms, which could help prevent the spread of this foodborne pathogen	MIC	n.a.	n.a.	1.000 μg/mL	n.a.	[99] Elgamoudi (2021).
Lemon (*Citrus limon*)	To investigate the effectiveness of oils and vapors of EOs and their components against a number of common foodborne pathogens	MIC, survival, inhibition area	Limonene	Cabbage leaf, chicken skin	18.3 ± 3 mm	n.a.	[107] Fisher (2006).
Lemon grass (*Cimbopogon citrates*)	Evaluation of twelve essential oils against *C. j.* in vitro and on food.	Inhibition zone, MIC, time-kill assay	n.a.	BHI broth, beef, and chicken	12.3–16.6 ± 0.7 mm 3.780–11.970 µg/mL	n.a.	[106] Rattanachaikunsopon (2010).
Myrtle (*Lemon myrtle*)	To find out how effective EOs and terpenoids are at inhibiting *C. jejuni* growth	Disk diffusion assay, MIC—microdilution, in vitro fermentation assay	Citral, Geranial	MH agar plate, 96-well plates	10.000 µg/mL, 90 mm	n.a.	[102] Kurekci (2013).
Manuka (*Leptospermum* scoparium)	To find out how effective EOs and terpenoids are at inhibiting *C. jejuni* growth	Disk diffusion assay, MIC—microdilution, in vitro fermentation assay	Citral, Citronellal	MH agar plate, 96-well plates	10.000 µg/mL, 90 mm	n.a.	[102] Kurekci (2013).
Mentha (*Mentha pulegium* L.)	EO can enhance the effectiveness of antibiotics/biofilm-forming capacity/	Agar-well diffusion assay, MIC, MBC—microdilution, biofilm production	Pulegone (40.98%), Menthone (21.164%)	LB broth, 96-well polystyrene microtiter plate	2.250 µg/mL, 17–80 mm	2 250 µg/mL	[88] El Baaboua (2022).
Mastic (*Pistacia lentiscus*)	Pathogen reduction, lipid oxidation, and sensory freshness	Disk diffusion assay, MIC, sensory analysis	1.8-cineole, β-Myrcene	96-well plates, chicken meat,	25.3 ± 1.52	0.6 ± 0.02	[101] Djenane (2012).
Orange (*Citrus × sinensis*)	EO can inhibit the growth of two important foodborne pathogens under laboratory conditions	Disk diffusion	n.a.	BHI broth, Charcoal Cefoperazone Deoxycholate agar	16–19 mm	n.a.	[95] Thanissery (2014).
Olive (*Olea europaea* L.)	Anti-adhesion effects of EO against *C. j.* on polystyrene surfaces and intestinal epithelial cells	Cytotoxicity, anti-adhesion test	n.a.	PSI cl1, H4 cells	n.a.	n.a.	[111] Šikić (2016).
Oregano (*Origanum compactum Benth*)	EO can inhibit resistant *Campylobacter* bacteria and prevent them from forming protective biofilms	Agar-well diffusion assay, MIC, MBC—microdilution, biofilm formation	Carvacrol (43.584%), *p*-cymene (18.587%) Thymol (10.331%)	LB broth, 96-well polystyrene microtiter plate	576–2.288 µg/mL 15–80 mm	576–2.288 µg/mL depend on the surface	[88] El Baaboua (2022).
Oregano (*Origanum minutiflorum*)	Evaluation of the in vitro antimicrobial activity of the EO against ciprofloxacin-resistant *C*. species	Agar-well diffusion, MIC	Carvacrol, *p*-cymene	BHI broth, Tryptone Soy Agar	7.8–800 μg/mL	n.a.	[96] Aslim (2008).
Oregano (*Origanum vulgare*)	Evaluation of the antibacterial activity of 5 EOs in beef meatballs	Agar disk diffusion, MIC, MBC, sensory analysis	Carvacrol	MH broth, Tryptic soy agar	1.162–2.325 μg/mL	n.a.	[92] Pesavento (2015).
Oregano (*Origanum vulgare*)	Evaluating how these essential oils can fight harmful microbes and prevent oxidation, which is important for food safety and preservation	Agar-well diffusion, MIC, MBC, antioxidant activity	Carvacrol, *p*-Cymene	MH broth, CCDA	5.65 μg/mL, 16.8–25.3 ± 2.9 mm	5.65 μg/mL	[75] Mutlu-Ingok (2021).
Peppermint (*Mentha x piperita*)	To investigate how peppermint essential oil affects the stress response and virulence potential of *C. j.*	MIC, MBC, SEM, motility assay, qRT-PCR, 2D SDS-PAGE, LC-MS, GC, TLC-DB	Menthol. Menthone, Isomenthone	BHI agar plates and medium	28–32 mm, 100 μg/mL	400 μg/mL	[53] Kovács (2019).
Rosmary (*Rosmarinus officinalis*)	EO can inhibit the growth of two important foodborne pathogens under laboratory conditions	Disk diffusion	n.a.	BHI broth, CCDA	11–17 mm	n.a.	[95] Thanissery (2014).
Rosmary (*Rosmarinus officinalis*)	Evaluation of the antibacterial activity of 5 EOs in beef meatballs	Agar disk diffusion, MIC, MBC, sensory analysis	1.8-cineol, Camphor, α-pinene	MH broth, Tryptic soy agar	910 μg/mL	n.a.	[92] Pesavento (2015).
Rosmary (*Rosmarinus officinalis* L.)	EO can inhibit resistant *Campylobacter* bacteria and prevent them from forming protective biofilms	Agar-well diffusion assay, MIC, MBC—microdilution, biofilm inhibition	1.8-cineole, α-pinene, Camphor	LB broth, 96-well polystyrene microtiter plate	>18.800 µg/mL–2.350 µg/mL 14–70 mm	n.a.	[88] El Baaboua (2022).
Sage (*Salvia officinalis*)	Evaluation of the antibacterial activity of 5 EOs in beef meatballs	Agar disk diffusion, MIC, MBC, sensory analysis	α-thujone, 1.8-Cineole	MH broth, Tryptic soy agar	5.700 μg/mL	n.a.	[92] Pesavento (2015).
Sweet inula (*Inula graveolens*)	Pathogen reduction, lipid oxidation, and sensory freshness	Disk diffusion assay, MIC, sensory analysis	Bornyl acetate, Borneol	96-well plates, chicken meat,	53.3 ± 9 μg/mL,	0.2 ± 0.02 μg/mL,	[101] Djenane (2012).
Sweet orange (*Citrus sinensis*)	To investigate the effectiveness of oils and vapors of EOs and their components against a number of common foodborne pathogens	Inhibition area, MIC, survival	Limonene	Cabbage leaf, chicken skin	0 mm, 36.000 μg/mL	n.a.	[107] Fisher (2006).
Tea tree (*Melaleuca alternifolia*)	Simple in vitro efficacy testing of tea-tree EO and its components	Disk diffusion assay, broth microdilution assay, in vitro fermentation assay	Terpinen-4-ol, γ-Terpinene	MH agar plate, 96-well plates	0.001%, 26.7–29.3 ± 0.7 mm	n.a.	[102] Kurekci (2013).
Thyme (*Thymus vulgaris*)	Evaluating how these essential oils can fight harmful microbes and prevent oxidation, which is important for food safety and preservation	Agar-well diffusion, MIC, MBC, antioxidant activity	Thymol, *p*-Cymene	MH broth, CCDA	5.65 μg/mL, 16.8–25.3 ± 2.9 mm	5.65 μg/mL	[75] Mutlu-Ingok (2021).
Thyme (*Thymus vulgaris*)	Testing EO at different conditions on *C. jejuni* in chicken burger and chicken shawerma	Inhibition zone	n.a.	MH broth ,chicken	20 mm (27.000 μg/mL)	n.a.	[109] Elsharawy (2018).
Thyme (*Thymus vulgaris*)	EO can inhibit the growth of two important foodborne pathogens under laboratory conditions	Disk diffusion, MIC, MBC	n.a.	BHI broth, Charcoal cefoperazone deoxycholate agar	no visible growth on the plate	n.a.	[95] Thanissery (2014).
Thyme (*Thymus vulgaris*)	To develop and evaluate electrospun thyme essential oil/gelatin nanofibers as active packaging material to inhibit *C. j.* in chicken	TEM, SDS-PAGE, TCPNs embedded gelatin nanofibers, FTIR, SEM, AFM	n.a.	Chicken	25.10 mm	n.a.	[112] Lin (2018).
Thyme (*Thymus vulgaris* L.)	Testing anti-adhesion effects of EO against *C. j.* on polystyrene surfaces and intestinal epithelial cells	Cytotoxicity, anti-adhesion test	n.a.	PSI cl1,H4 cells	n.a.	n.a.	[111] Šikić (2016).
Thyme (*Thymus vulgaris*)	Testing whether certain essential oils can inhibit the growth of *C. jejuni*, a common cause of foodborne illness	Abiotic surface	n.a.	Chicken	n.a.	n.a.	[90] Salem (2019).
Thyme (*Thymus vulgaris*)	Evaluation of the antibacterial activity of EO in beef meatballs	Agar disk diffusion, MIC, MBC, sensory analysis	*p-cymene*	MH broth, Tryptic soy agar	1.150–2.300 µg/mL	1.150 µg/mL	[92] Pesavento (2015).
Turmeric (*Curcuma longa*)	Evaluation of twelve essential oils against *C. j.* in vitro and on food.	Inhibition zone, MIC, time-kill assay	n.a.	BHI broth, beef, and chicken	15.7–22.4 ± 0.5 mm 0.06–0.83 *v*/*v*%	n.a.	[106] Rattanachaikunsopon (2010).
Wild carrot (*Daucus carota* L.)	To find out which compounds in carrot essential oil inhibit the growth of *C. j.* and understand how they work	MIC	β-bisabolene, Elemicin, α-pinene	MH agar	125–200 μg/mL	n.a.	[104] Rossi (2007).
Winter savory (*Satureja montana*)	Pathogen reduction, lipid oxidation, and sensory freshness	Disk diffusion assay, MIC, sensory analysis	Carvacrol, *p*-cymene, Thymol	96-well plates, chicken meat	25.8 ± 0.2	0.6 ± 0.02	[101] Djenane (2012).

## 6. Essential Oils Modulating Biofilm Formation of *Campylobacter jejuni*

Biofilm formation plays a significant role in the infectious strategy of microorganisms. Biofilms protect bacteria from adverse environmental effects, such as dehydration and phagocytosis, and also concentrate nutrients [113]. Several *C. jejuni* isolates exhibit strong biofilm formation on both biotic and abiotic surfaces [114]. The formation of *C. jejuni* biofilms is influenced by various external factors, including the environment, oxygen saturation, metabolites, and nutrient availability. From a bacterial perspective, the roles of motility, chemotaxis, the flagella, and quorum sensing are crucial aspects of biofilm formation [115]. Intrinsic factors in biofilm formation include flagella-coding genes, and the PPK1 and PPK2 kinases, which play a key role in stress responses, colonization, and virulence. The presence of the PhosX regulatory system, which evolved to tightly regulate phosphate homeostasis, enables bacteria to survive and adapt in nutrient-limited environments [116]. A nutrient-deficient medium promotes biofilm formation [85], as does oxygen saturation [117]. *Campylobacter* can form a biofilms on both biotic surfaces, such as meat, fruit, and vegetables [118,119,120], as well as on abiotic surfaces, such as steel and plastic, which are frequently used in the food industry during meat processing. Biofilms formed on these surfaces can lead to human infections [121], which is why recent studies have focused on the potential of EOs to inhibit and degrade biofilms [87,89,122].

The essential oil *Lavandula angustifolia* was found to hinder the formation of biofilms by *C. jejuni* isolates by downregulating the genes responsible for the capsule, an accessory element that plays a crucial role in adhesion [89]. This capsule serves multiple functions, but not all *C jejuni* isolates produce it—only those that possess cps genes, such as *kpsM*, *kpsT* and *kpsC*. The role of the capsule in biofilm formation has been the subject of extensive research, with several studies confirming that mutants lacking a capsule exhibit reduced biofilm-forming potential [123,124,125]. It has also been demonstrated that this EO can break up mature biofilms that have already formed [89]. Although the antibiofilm effect of *L. augustifolia* could be detected in the distilled essential oil of the flowers, the pre-distillation extract, and the ethanolic extract of the waste material left after distillation, the ethanolic extract of the flowers before distillation was found to be the most effective [89]. Other research suggests that *Lavandula × intermedia* and related lavender species effectively reduce *C. jejuni* biofilm formation. Ramić et al. (2021) [89] also revealed that ethanolic extracts reduce intercellular signaling between the eukaryotic cell and *C. jejuni*, thereby affecting adhesion. Similar to lavender, coriander has also been shown to inhibit *C. jejuni* biofilm formation [126]. In this study, the authors demonstrated that linalool, a major component of lavender, alone had a biofilm-inhibitory effect in the applied concentration (0.5× MIC: 0.38  ±  0.15, 1× MIC, 2× MIC, OD: 0.043  ±  0.005). The MIC value for lavender EO was 250 ± 60 µg/mL. Biofilm degradation was performed using the MIC value and its twofold concentration. At 2× MIC, the surface coverage was described as “almost zero” [89]. In contrast, for coriander, the MIC value was higher at 500 µg/mL, although a different bacterial strain was tested. Biofilm degradation was assessed at 0.5×, 1×, 2×, and 4× MIC values for each strain. A significant correlation was observed between increasing concentrations of essential oil and biofilm-degrading activity. At 0.5× MIC, biofilm inhibition was approximately 35%, while at 4× MIC, inhibition ranged from 43% to 77% depending on the strain. Lavender EO therefore appears to be more effective, although it was only tested against one bacterial strain [126].

## 7. Essential-Oil-Based Control of *Campylobacter jejuni* in Food

Today, studies are attempting to translate the recently gained in vitro information into practice. This is highly reasonable, given that *C. jejuni* is a common and significant foodborne pathogen and there is a demand for natural control solutions. This is one reason for the number of studies analyzing the applicability of EOs as potential anti-campylobacter disinfectants in and on different food matrices, as well as on different surfaces such as stainless steel—a common material in slaughterhouse environments where cross-contamination among slaughtered animals can occur [127,128]. From a statistical point of view, chicken meat is the main focus of these studies (Table 2). Accordingly, 71% of surface cleaning experiments were conducted on chicken meat [101,106,129,130,131], while cattle (10%) [106,132] were the second and third most common model organisms, followed by ducks (5%) [133].

Experiments were carried out on the aforementioned food matrices with different essential oils at temperatures ranging from 3 to 42 °C [90,101,108]. During the experiments, different procedures were used for contamination, such as submerging the meat in a bacterium suspension [108] or dropping a certain amount of a bacterium suspension with a known CFU count onto the meat surfaces [130]. The germ counts applied to the meat surfaces were fairly broad, spanning from 10^3^/mL [108], to 10^8^/mL [101]. Essential oils such as coriander [90], thyme [90], lime [130], oregano [129], chrysanthemum [108], and bay leaf [101] were applied at different concentrations, typically at 4.000 µg/mL [90].

There were also differences among the different treatment methods. Temperature has a practical relevance as it has been proven to affect the efficacy of several EOs. In the case of 2.0, 3.0, 4.0, 5.0, and 6.0 mg/mL chrysanthemum essential oil (*Helichrysum italicum*) [108] when applied to chicken meat at 25 °C and 37 °C, the efficacy ranged from 28–63% for the tested isolates. At lower temperatures, such as 4 °C and 12 °C, the efficacy was very convincing: 100%. Interestingly, this temperature dependent efficacy could be observed in the case of *Listeria monocytogenes* isolates. This was due to cellular changes in the organism, as most EOs were ineffective at 14 °C despite being active at low (4 °C) and high (37 °C) temperatures [134]. Validating the applied concentration is an important aspect with economic significance. Using two different concentrations of thyme and coriander essential oils (1% and 2%), the initial germ count (3.8 × 10^7^) could be drastically lowered at 4 °C. After six days of incubation, thyme EO reduced the initial germ count on the surface of chicken by 97.27% and 99.99%, while coriander EO reduced the initial germ count by 85% and 96.27%. In this experiment, 2% thyme oil achieved nearly 100% effectiveness after 6 days [90].

The efficacy of the Dayak onion (*Eleutherine americana*) was compared in three ways: (i) the meat was immersed in the extract for 30 min, (ii) the meat was mixed with the extract, and (iii) the extract was frozen −20 °C for 24 h. For each experiment, the chicken samples were treated with extracts of various concentrations. A quantity of 10 mL of extract at various concentrations was added to 100 g of chicken meat that had previously been inoculated with *C. jejuni*. The applied extract concentrations were 0.5, 1, 2, 4, and 8 mg/mL. Using the “dipping method” a reduction of approximately 2 logs in the number of *C. jejuni* ATCC 33560 on the chicken meat surface could be achieved by day 6 if the most concentrated extract (8 mg/mL) was used. Using the “mixing” method, a 1 log reduction could be achieved by day 7 if a 4 mg/mL extract concentration was used. Using a combination treatment involving a 4 mg/mL and 8 mg/mL extract followed by −20 °C, a reduction of approximately 2 logs could be achieved over six days [131]. The effects of three marinades containing different combinations of EOs, such as lemon juice, thyme oil and black pepper (M1); lime juice, lemongrass oil, and chili paste (M2); and olive oil, oregano oil, basil oil, and garlic paste (M3), were tested against *C. jejuni* on marinated chicken thighs stored aerobically at 4 °C. The first two marinades effectively reduced the initial concentration from 6 log CFU/g to 2 log CFU/g [135]. Different bacterial isolates belonging to the same species, i.e., *C. jejuni*, can exhibit different sensitivities to various EOs [131].

In crucial cases, a straightforward method is to combine EOs to achieve a broad and firm antimicrobial effect that covers the entire *C. jejuni* species, as well as a broader range of foodborne bacteria such as *Salmonella*, *Listeria monocytogenes*, certain *E. coli* pathotypes and *Yersinia enterocolitica* [136,137,138]. To achieve this, a combination of different essential oils can be used. Some researchers wanted to use a mixture of essential oils [105,137]. Some applied the oils directly while others used an emulsion method [90]. One of the most effective essential oils was lime oil, which was applied directly to the surface of raw chicken and incubated at 4 °C. The CFU was reduced from 5 log CFU/mL to 1 log CFU/mL [130].

Djenane and colleagues (2012) [101] used four different plant essential oils in their experiments: *Inula graveolens*, *Laurus nobilis*, *Pistacia lentiscus*, and *Satureja montana*. Their experiments proved that *I. graveolens* was the most effective EO against *C. jejuni*, using both the agar disk diffusion method and the minimum inhibitory concentration (MIC) method. The inoculated meat samples were stored in a microaerobic atmosphere in a refrigerated environment at 3 ± 2 °C, and bacterial growth was regularly monitored. The application of essential oils significantly reduced the growth of *C. jejuni* in the treated meat samples. While the bacterial count in the control samples increased by up to 8 log_10_ CFU/g during storage, the bacterial load in the samples treated with essential oils decreased by between 0.7 and 4.7 log_10_ CFU/g, remaining significantly lower throughout the storage period. *Inula graveolens* EO in particular proved to be effective, with a minimum inhibitory concentration (MIC) of around 2 µL/mL (2 mg/mL). In addition to its antibacterial effect, the treatment also reduced lipid oxidation in the meat, thereby extending its shelf life and improving its sensory properties, particularly with regard to odor preservation. This antioxidant effect is key to maintaining meat quality [101].

Another biotic test surface that was recently tested was a cabbage leaf [107]. In the study, the bacteria were inoculated at an initial load of 6–7 log_10_ CFU/mL, followed by the application of essential oils at different concentrations: 0.1%, 0.5%, and 1.0% (*v*/*v*). Then, essential oils were applied at various concentrations: 0.1%, 0.5%, and 1.0% (*v*/*v*). The treatment duration ranged from 2 h to 24 h, depending on the experimental conditions.

The results showed that the antibacterial effect of the essential oils was concentration- and time-dependent. At the lowest concentration of 0.1%, the *C. jejuni* population decreased by approximately 1–2 log_10_ units within 24 h. At the medium concentration of 0.5%, the reduction was more pronounced at around 3 log_10_ units. At the highest concentration of 1.0%, a decrease of 4–5 log_10_ units was observed within the same timeframe.

The efficacy was further enhanced by the complex composition of the essential oils, with the components limonene, citronellal, and linalool synergistically contributing the bactericidal effect. However, environmental factors significantly influenced the results. In vitro tests using pure solutions demonstrated the most potent effect, whereas antibacterial activity diminished in food matrices such as meat or milk, typically resulting in a reduction of 1–3 log_10_ units. This decrease was primarily due to binding interactions and diffusion barriers within the food composition that limited the availability of the essential oils’ active compounds to bacteria.

When essential oils are applied to food, the question arises as to whether they affect the texture and flavor when consumed. This is why an increasing number of sensory testing experiments involving meat are now being conducted to examine its texture and sensory properties [92,101]. Experiments aimed at targeting daily challenges associated with *C. jejuni* should be guided by daily food preservation practices.

**Table 2:** Anti-Campylobacter effect of essential oils in different food matrices.

Food	Aim of the Study	Method	Applied EO	*C. jejuni* CFU Before Treatment	After Treatment	Tempe-Rature	References
Chicken minced	Testing antimicrobial effects of selected essential oils against *C. jejuni* in poultry meat stored in polyethylene bags	Polyethylene bag	9 mg/mL thyme oil	25 × 10^6^ ± 1.02 × 107 CFU/mL	7.3 × 10^5^ ±3.6 × 10^5^ CFU/mL (6th day)	4 °C	[90] Salem (2019).
Chicken minced	Testing antimicrobial effects of selected essential oils against *C. jejuni* in poultry meat stored in polyethylene bags	Polyethylene bag	18 mg/mL thyme oil	25 × 10^6^ ± 1.02 × 107 CFU/mL	2.5 × 10^3^ ± 1.2 × 10^3^ CFU/mL (6th day)	4 °C	[90] Salem (2019).
Chicken minced	Testing antimicrobial effects of selected essential oils against *C. jejuni* in poultry meat stored in polyethylene bags	Polyethylene bag	9 mg/mL coriander oil	25 × 10^6^ ± 1.02 × 107 CFU/mL	3.8 × 10^6^ ±2.1 × 10^5^ CFU/mL (6th day)	4 °C	[90] Salem (2019).
Chicken minced	Testing antimicrobial effects of selected essential oils against *C. jejuni* in poultry meat stored in polyethylene bags	Polyethylene bag	18 mg/mL coriander oil	25 × 10^6^ ± 1.02 × 107 CFU/mL	9.5 × 10^5^ ±1.2 × 10^5^ CFU/mL (6th day)	4 °C	[90] Salem (2019).
Chicken skin	Testing effectiveness of fruit extracts in reducing *C. jejuni* on poultry skin.	Surface	Lime 2 ± 0.1 mg/mL	1 × 10^5^ CFU/mL	1 × 10^1^ CFU/mL	4 °C	[130] Valtierra- Rodríguez (2010).
Chicken meat	Testing essential oil against *C. jejuni* in microaerobic-packaged chicken	Packaged	*Inula graveolens* (2× MIC)	8.14 log_10_ CFU/g	1.2 log_10_ CFU/g	3 ± 2 °C	[101] Djenane (2012).
Chicken meat	Testing essential oils against *C. jejuni* in microaerobic-packaged chicken	Packaged	*Laurus nobilis* (2× MIC)	8.14 log_10_ CFU/g	1.99 log_10_ CFU/g	3 ± 2 °C	[101] Djenane (2012).
Chicken meat	To evaluate essential oils against *C. jejuni* in microaerobic-packaged chicken	Packaged	*Pistacia lentiscus* (2× MIC)	8.14 log_10_ CFU/g	2.2 log_10_ CFU/g	3 ± 2 °C	[101] Djenane (2012).
Chicken meat	To evaluate essential oils against *C. jejuni* in microaerobic-packaged chicken	Packaged	*Satureja montana* (2× MIC)	8.14 log_10_ CFU/g	2.2 log_10_ CFU/g	3 ± 2 °C	[101] Djenane (2012).
Chicken meat	Testing pulsed electric fields and antimicrobial compounds, alone and in combination, in inactivating *Campylobacter jejuni* in liquids and raw chicken.	Mix (PEF and Oregano), Oregano alone	Oregano	4.41± 0.20 log_10_ CFU/g	0.2–1.9 log_10_	42 °C	[129] Clemente (2020)
Chicken breast	Testing efficacy of coriander oil in controlling *C. jejuni* during storage	Bag	Coriander oil (0.9; 2.25; 4.5 mg/mL)	5 log CFU/g	0.05 *v*/*v*%: 0 log CFU	4 °C	[106] Rattanachaikunsopon (2010)
Chicken breast	Testing efficacy of coriander oil in controlling *C. jejuni* during storage	Bag	Coriander oil (0.9; 2.25; 4.5 mg/mL)	5 log CFU/g	0.05 *v*/*v*%: 0 log CFU	32 °C	[106] Rattanachaikunsopon (2010)
Beef lean	Testing efficacy of coriander oil in controlling *C. jejuni* during storage	Bag	Coriander oil (0.9; 2.25; 4.5 mg/mL)	5 log CFU/g	0.05 *v*/*v*%: 0 log CFU	4 °C	[106] Rattanachaikunsopon (2010).
Beef lean	Testing efficacy of coriander oil in controlling *C. jejuni* during storage	Bag	Coriander oil (0.9; 2.25; 4.5 mg/mL)	5 log CFU/g	0.05 *v*/*v*%: 0 log CFU	32 °C	[106] Rattanachaikunsopon (2010).
Duck meat	Testing antimicrobial effectiveness and practical application of casein/cinnamon oil nanospheres against *C. jejuni* in duck meat	Mixed, Nanospeheres	Cinnamon essential oil and kazein	4.30 logCFU/g	0.86 log CFU/g	4 °C	[133] Cui (2021).
Duck meat	Testing antimicrobial effectiveness and practical application of casein/cinnamon oil nanospheres against *C. jejuni* in duck meat	Mixed, Nanospeheres	Cinnamon essential oil and kazein	4.30 logCFU/g	2.46 logCFU/g	25 °C	[133] Cui (2021).
Chicken breast	Testing liposome characteristics, antimicrobial efficacy, and practical application on chicken meat against *C. jejuni*	Triple-layer liposomes	Chrysanthemum essential oil 25, 50, 75 and 100 µg/mL	3.2 log CFU/mL	0 log CFU/mL	4 °C	[108] Lin (2019).
Chicken breast	Testing liposome characteristics, antimicrobial efficacy, and practical application on chicken meat against *C. jejuni*	Triple-layer liposomes	Chrysanthemum essential oil 25, 50, 75 and 100 µg/mL	3.2 log CFU/mL	0 log CFU/mL	12 °C	[108] Lin (2019).
Chicken breast	Testing liposome characteristics, antimicrobial efficacy, and practical application on chicken meat against *C. jejuni*	Triple-layer liposomes	Chrysanthemum essential oil 25, 50, 75 and 100 µg/mL	3.2 log CFU/mL	1.2 log CFU/mL	25 °C	[108] Lin (2019).
Chicken breast	Testing liposome characteristics, antimicrobial efficacy, and practical application on chicken meat against *C. jejuni*	Triple-layer liposomes	Chrysanthemum essential oil 25, 50, 75 and 100 µg/mL	3.2 log CFU/mL	2.3 log CFU/mL	37 °C	[108] Lin (2019).
Chicken drumstick	Survival rates of different CFUs of *C. jejuni* were revealed in mixed chicken/gram	Mixed	Lemon juice, Thyme oil and black pepper (450 µg/mL from thyme)	6.1 log CFU/g	1 log CFU/g	4 °C	[135] Marmion (2023).
Chicken drumstick	Survival rates of different CFUs of *C. jejuni* were revealed in mixed chicken/gram	Mixed	Lime juice, Lemongrass oil and chilli paste (9 mg/mL from lemongrass)	6.1 log CFU/g	2.6 log CFU/g	4 °C	[135] Marmion (2023).
Chicken drumstick	Survival rates of different CFUs of *C. jejuni* were revealed in mixed chicken/gram	Mixed	Olive oil, Oregano oil, Basil oil and garlic paste (9 mg/mL from basil, 4.5 mg/mL from oregano)	6.1 log CFU/g	5.5 log CFU/g	4 °C	[135] Marmion (2023).
Chicken meat	Survival rates of different CFUs of *C. jejuni* were revealed in mixed chicken/gram	Dipping chicken meat in the extract for 30 min	*Eleutherine americana* bulb 4 mg/mL, 8 mg/mL	6 log_10_ CFU/g	3–5.5 log_10_ CFU/g	4 °C	[131] Musthafa (2021).
Chicken meat	Survival rates of different CFUs of *C. jejuni* were revealed in mixed chicken/gram	Mixing the extract with chicken meat	*Eleutherine americana* bulb 4 mg/mL, 8 mg/mL	6 log_10_ CFU/g	2–5.8 log_10_ CFU/g	4 °C	[131] Musthafa (2021).
Chicken meat	Survival rates of different CFUs of *C. jejuni* were revealed in mixed chicken/gram	Combination of the extract with short-term freezing at −20 °C for 24 h.	*Eleutherine americana* bulb 4 mg/mL, 8 mg/mL	4.8 log_10_ CFU/g	2–3.2 log_10_ CFU/g	4 °C	[131] Musthafa (2021).

## 8. Conclusions

Despite the still-moderate number of studies, the anti-campylobacter effects of certain essential oils, such as clove, oregano, thyme, rosemary, lavender, sage, tea tree, etc. in vitro and in food matrices in practice-oriented systems confirms that EOs have potential in food preservation. These promising results have contributed to develop prevention procedures (EO mixtures) that target not only *C. jejuni* but also other foodborne pathogens. Future studies should focus on establishing such mixtures and addressing their stability and sensory properties to ensure an effective disinfectant for the food industry. Another progression route of development is to identify active compounds and use them for decontamination procedures, either against different bacteria, and hence make the prevention more target-bacterium-oriented.

## Data Availability

No new data were created or analyzed in this study. Data sharing is not appliable to this article.

## Abbreviations

The following abbreviations have been used in this manuscript:

AFM	Atomic Force Microscopy
BHI	Brain–Heart Infusion
CCDA	Charcoal Cefoperazone Deoxycholate Agar
DPPH	2,2-Diphenyl-1-picrylhydrazyl free radical assay
GBS	Guillain-Barré syndrome
CFU	Colony-Forming Unit
EO	Essential Oil
FTIR	Fourier-Transform Infrared Spectroscopy
GC	Gas Chromatography
LB	Luria Bertani
LC-MS	Liquid Chromatography–Mass Spectrometry
MBC	Minimum Bactericide Concentration
MFS	Miller Fisher Syndrome
MH	Mueller Hinton
MIC	Minimum Inhibitory Concentration
PCR	Polymerase Chain Reaction
PAGE	Polyacrylamide Gel Electrophoresis
qPCR	Quantitative Polymerase Chain Reaction
SEM	Scanning Electron Microscopy
TEM	Transmission Electron Microscopy
TLC-DB	Thin-Layer Chromatography–Direct Bioautography
VBNC	Viable But Non-Culturable
